# Integration of Stemness Gene Signatures Reveals Core Functional Modules of Stem Cells and Potential Novel Stemness Genes

**DOI:** 10.3390/genes14030745

**Published:** 2023-03-18

**Authors:** Tânia Barata, Isabel Duarte, Matthias E. Futschik

**Affiliations:** 1 SysBioLab, Centre for Biomedical Research (CBMR), Universidade do Algarve, 8005-139 Faro, Portugal; 2 Center for Research in Health Technologies and Information Systems (CINTESIS), Universidade do Algarve, 8005-139 Faro, Portugal; 3 School of Biomedical Sciences, Faculty of Health, Derriford Research Facility, University of Plymouth, Plymouth PL6 8BU, UK; 4 MRC London Institute of Medical Sciences (LMS), Imperial College London, London W12 0NN, UK; 5 NOVA Medical School, Universidade NOVA de Lisboa, 1169-056 Lisbon, Portugal

**Keywords:** stemness, stem cells, gene signatures, data integration

## Abstract

Stem cells encompass a variety of different cell types which converge on the dual capacity to self-renew and differentiate into one or more lineages. These characteristic features are key for the involvement of stem cells in crucial biological processes such as development and ageing. To decipher their underlying genetic substrate, it is important to identify so-called stemness genes that are common to different stem cell types and are consistently identified across different studies. In this meta-analysis, 21 individual stemness signatures for humans and another 21 for mice, obtained from a variety of stem cell types and experimental techniques, were compared. Although we observed biological and experimental variability, a highly significant overlap between gene signatures was identified. This enabled us to define integrated stemness signatures (ISSs) comprised of genes frequently occurring among individual stemness signatures. Such integrated signatures help to exclude false positives that can compromise individual studies and can provide a more robust basis for investigation. To gain further insights into the relevance of ISSs, their genes were functionally annotated and connected within a molecular interaction network. Most importantly, the present analysis points to the potential roles of several less well-studied genes in stemness and thus provides promising candidates for further experimental validation.

## 1. Introduction

Stemness is the potential of stem cells for self-renewal and differentiation into one or more lineages. These inherent traits place stem cells in the core of complex biological processes including human development [1] and ageing [2]. Additionally, stem cells have attracted considerable interest in biomedicine, especially since the establishment of methods for induced pluripotency that have revolutionized this research field [3]. The generation of differentiated cell types from stem cells can help with studying degenerative disorders [3] and can provide the basis for cellular replacement therapies in regenerative medicine [1,3]. Furthermore, several studies have shown the crucial role of stem cells in the development of cancer by revealing similarities between stem cells and cancer cells, particularly regarding the activation of similar pathways as well as on the phenotypic level [1,4], therefore highlighting the importance of the stemness concept for cancer research.

There are numerous types of stem cells with different levels of plasticity, of which some of the best-studied are embryonic, neural, and hematopoietic stem cells. Despite their distinct functions, all these cell types share the fundamental property of stemness. Therefore, it has been tempting to postulate that stemness is the consequence of the activation (or repression) of specific molecular pathways, and thus it can be linked to a defined set of genes [5]. Indeed, the advent of genome-wide profiling technology led different groups to identify so-called stemness signatures comprised of genes whose activity is characteristic of a certain type of stem cells, or even of stem cells in general [6,7]. However, when the initial microarray-based stemness signatures were compared, only a small number of genes were found to be common [8]. Nevertheless, numerous other research groups have brought forward stemness signatures for various types of stem cells using different methods [9].

In the present study, we greatly extend earlier comparisons of stemness signatures by including more gene signatures for both mice and humans, and by covering a wider range of distinct stem cell types. Notably, we have also broadened the methodological techniques used to derive these stemness signatures. Gene sets were obtained not only from transcriptomics experiments, but also RNAi screens, curated pathway databases, and text-mining. Thus, a greater range of stem cell types, and methodological approaches provided the basis for this extensive stemness signature meta-analysis, enabling us to detect characteristic trends of genes associated with stemness.

Using this rich basis, we obtained integrated stemness signatures (ISSs), comprising genes most frequently found among individual studies. Such consolidated signatures add more confidence to the association of stemness with the included genes, and help to exclude false positives that could have compromised individual studies. Our analysis allows the pinpointing of genes that might have been overlooked in previous studies due to their low scoring, for example in differential expression analyses, but which are repeatedly associated with stemness across several studies. Finally, the defined ISSs were further functionally annotated, and an analysis of their protein interaction network was performed to detect distinct sub-clusters.

## 2. Materials and Methods

### 2.1. Individual Stemness Signatures

Gene sets compared in this analysis were retrieved from StemChecker (accessible at http://stemchecker.sysbiolab.eu, accessed on 16 January 2020) [10]. This freely available resource (developed by our lab), allows researchers to rapidly screen gene lists for the presence of stemness signatures that were manually curated from published literature or other relevant databases. These stemness signatures were classified into different categories based on the method used for their prediction: (i) Expression profiling that identifies sets of genes up-regulated in diverse stem cell types when compared to differentiated cells using transcriptomics (31 signatures); (ii) RNAi screens that use the read-out of reporter genes for pluripotency to assess the impact of genome-wide RNAi knock-downs (5 signatures); (iii) Literature curation that links genes to stem cells based on the reviewing of published literature (4 signatures) and (iv) Computational prediction where computational network analysis and text-mining databases are used to associate genes with stem cells (2 signatures). Furthermore, the stemness signatures were grouped into signatures for pluripotent, multipotent, unipotent, and mixed stem cell populations. The individual stemness signatures, together with the sources and groupings, are listed in Supplementary Table S1 [6,7,8,11,12,13,14,15,16,17,18,19,20,21,22,23,24,25,26,27].

### 2.2. Accessing Similarity among Stemness Signatures

To determine the similarity among different human or mouse stemness signatures, we generated clustered heatmaps based on the significance level of the overlap of genes between stemness signatures. The significance of the overlap was determined by the hypergeometric test as implemented in R [28] and adjusted for multiple hypothesis testing using the ‘Bonferroni’ correction. The universe of genes for each organism was defined by genes annotated in Gene Ontology (GO). Clustered heatmaps of log_10_ (adjusted *p*-value) of gene overlap and the dendrograms representing the Euclidean distance between stemness signatures were produced using the *heatmap.2* function from the *gplots* [29] package (Supplementary Figure S1). Colour schemes were derived using the *RColorBrewer* [30] package. The R Bioconductor packages *org.Hs.eg.db* [31] and *org.Mm.eg.db* [32] were used, respectively, for human and mouse gene annotation.

### 2.3. Deriving Integrated Stemness Signatures

To obtain ISSs, for both mouse and human, the genes were ranked according to how often they appear (i.e., their frequency) in mouse and human signatures, respectively. The two lists were then sorted based on their respective scores. To assess the likelihood of obtaining the observed scores by chance, we applied a randomization procedure. First, gene sets of the same size as the original stemness signatures were generated, but with randomly selected genes from the relevant universe of genes. Subsequently, we recorded the frequency of occurrence for each gene across the random gene sets. To obtain an empirical background distribution, we repeated this procedure 10^5^ times and calculated the likelihood of obtaining a certain score by chance. Comparing the empirical background distribution with the scores obtained for the ISSs provides an estimate of the false discovery rate for the different scores observed [33]. Scores equal to or larger than 4 for human sets or equal to or larger than 5 in mouse sets show empirical FDR lower than 1 × 10^−3^ (Supplementary Figures S2 and S3). Subsequently, thresholds for the score were chosen, so that at least 100 genes of each ranked list were included in the ISSs to have enough genes for robust functional enrichment analysis. Thus, genes with a score equal to or higher than 4 in the human gene list (FDR = 4 × 10^−4^) and equal to or higher than 7 in the mouse gene list (FDR = 1 × 10^−5^) were selected (Supplementary Tables S2 and S3). Similarly, we calculated the frequency that a gene is occurring in stemness signatures associated with pluripotency or multipotency (Supplementary Tables S2 and S3). To make the different scores more comparable, they were optionally normalized by division of the maximum possible score (i.e., the number of signatures either associated with pluripotency or multipotency.) The R packages used to identify orthologs between mouse and human ISSs were *DBI* [34] and *hom.Hs.inp.db* [35]. To obtain the number of publications associated with genes in a specific context, the *entrez_search* function of the *rentrez* package [36] was used. For each gene included in the ISSs, a search was performed in PubMed to gather the number of publications where the gene name co-occurred with the term *stem cell(s)*, either in the full article or in the title or abstract.

### 2.4. Functional Analysis of Integrated Stemness Signatures

For functional enrichment analysis based on Gene Ontology (GO) [37,38], the hypergeometric test implemented on the *GOstats* [39] package was applied. GO terms were mapped to their corresponding gene identifiers using org.Hs.egGO2ALLEGS and org.Mm.egGO2ALLEGS objects of the R packages *org.Hs.eg.db* [31] and *org.Mm.eg.db* [32], respectively. Since the hierarchical GO structure often results in the detection of a large number of dependent terms as significant, a conditional algorithm implemented in GOstats was used to reduce the number of significant GO terms. This algorithm uses the structure of the GO graph to estimate for each term whether there is evidence beyond the one provided by the term’s children to call the term in question as being statistically overrepresented [39]. For this purpose, a cut-off *p*-value of 0.05 was set. For detecting enrichment in Reactome pathways [40], we used the *ReactomePA* package [41]. The *p*-value was adjusted for multiple testing using the Benjamini–Hochberg (‘FDR’) correction method as implemented in R. GO terms and pathways were considered significantly enriched when the corresponding adjusted *p*-value was below 0.05. The gene universe was composed of all human or mouse genes mapped to biological processes, molecular functions, cellular components, or Reactome pathways depending on the analysis performed. The Bioconductor package biomaRt [42] was used for this analysis.

### 2.5. Network Analysis of Integrated Stemness Signatures

Interactions for proteins that correspond to genes with minimum scores of 3 and 6 for the human (FDR = 1 × 10^−3^) and mouse ranked lists (FDR = 1 × 10^−5^), respectively, were retrieved from STRING [43] to provide a network context for the genes. Only interactions between the queried proteins (in the 1st shell) with a high confidence score (at least 0.7) based on experiments and database evidence were extracted together with corresponding confidence scores (combined scores in STRING). For the visualization of the network, the *Edge-weighted Spring Embedded* layout weighted by the confidence scores was applied in Cytoscape [44]. Nodes without interactions were excluded. To identify protein clusters we used the Cytoscape app ClusterOne [45] weighted by interaction confidence scores. Then, nodes of each significant cluster (with *p*-value < 0.05) were arranged in circles according to their betweenness centrality with Cytoscape *Attribute circle* layout. For humans, nodes that did not link to the main network were excluded before the clustering analysis. For mice, nodes that did not interact with the main network and were part of a small network (with less than three nodes) were excluded before the clustering analysis. *Average stemness* scores, corresponding to the mean of the stemness scores of the genes comprising a cluster, were calculated for each cluster (Supplementary Tables S4 and S5). Clusters were ranked according to the significance of the clustering (*p*-value rank column of Supplementary Tables S4 and S5) and the average stemness score (Average Stemness score rank column of Supplementary Tables S4 and S5). Pathway and GO enrichment analysis was carried out as previously described for the ISSs.

## 3. Results and Discussion

Forty-two individual stemness signatures (21 gene sets for mice and 21 for humans) based on transcriptomics experiments, RNAi screens, curated pathway databases, as well as computational and text-mining studies were collected (Supplementary Table S1). A diverse set of stem cell types was analysed. Signatures for well-studied stem cell types, such as embryonic or induced pluripotent, hematopoietic, neuronal, mesenchymal, and cancer stem cells were represented, as well as stemness signatures for less studied types, such as epithelial, intestinal, hair bulge, and spermatogonial stem cells (Supplementary Table S1). Altogether, these cover a total of 3602 unique human genes (Supplementary Table S2) and 6390 unique mouse genes (Supplementary Table S3).

Overall, the majority of stemness signatures showed significant overlap. In total, 119 of the 210 pairwise comparisons for human, and 148 of the 210 comparisons for mouse signatures led to the detection of significant overlap, with an adjusted *p*-value < 0.05. To examine the influence of biological and experimental methodology factors on the similarity between stemness signatures, we performed a pairwise comparison of stemness signatures’ genes, and subsequently clustered the stemness signatures based on the significance of the overlap. Our examination of the resulting cluster structures indicates that both stem cell origin and methodology play a role (Supplementary Materials: *Influence on stem cell origin and methodology on individual stemness signatures* and Supplementary Figure S1).

### 3.1. Integration of Stemness Signatures

As shown above, the experimental methodology and biological features are a confounding factor in individual stemness signatures, and therefore it is difficult to judge whether the genes included in those signatures are truly functionally relevant in the context of stem cell biology, or if they are false positives. If such genes, however, appear repeatedly in stemness signatures that were independently obtained, the statistical likelihood that they are false positives reduces [46]. Accordingly, to reduce the influence of the experimental methodology as a confounding factor, in this study we identified genes consistently associated with stemness across distinct individual signatures despite the different experimental setups applied and stem cell types tested.

Although we did not find any gene common to all stemness signatures, we observed several genes reoccurring in different signatures. Thus, we sought to obtain an ISS for humans and another for mice where genes were ranked according to their frequency in individual studies. First, scores corresponding to the number of occurrences in individual stemness signatures were calculated for each gene and used to rank genes (Supplementary Tables S2 and S3). To assess the significance of scores, a randomization procedure was carried out to estimate the probability of observing a specific score by chance, as described in *Materials and Methods*. Then, human genes with a minimum score of four, and mouse genes with a minimum score of seven, were assigned to the respective ISSs, as these scores showed high significance when compared to the random background distribution, i.e., FDR < 1 × 10^−3^ (Supplementary Figures S2 and S3). Resulting ISSs comprised the top 164 genes, corresponding to 4.55% of all ranked genes for humans; and the top 115 genes, corresponding to 1.8% of all ranked genes for mice.

**Figure 1:**
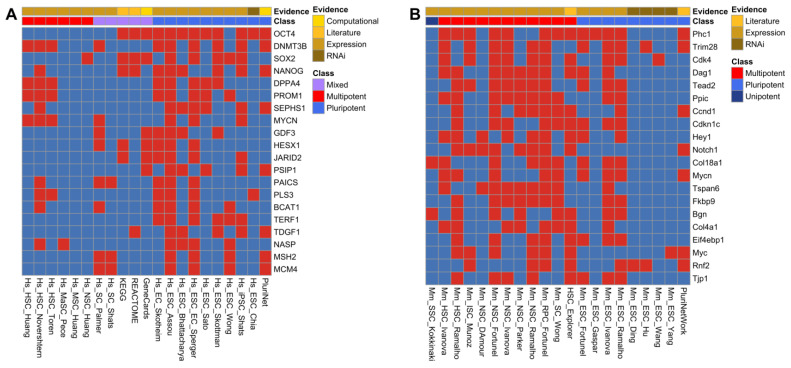
Association of top-scoring genes with stemness signatures. The checkerboards display the association (indicated by red) of genes (rows) with human (**A**) and mouse (**B**) stemness signatures. The stemness signatures were classified into signatures for pluripotency, multipotency, unipotency or of mixed potency based on the stem cell types (Supplementary Table S1). Furthermore, the underlying evidence for the stemness signatures is indicated (expression, RNAi, literature curation, computational derivation).

The master transcription factor genes for pluripotency, i.e., *NANOG*, *SOX2,* and *POU5F1* (the last one encoding OCT4), ranked at the top of the human ISS (Figure 1A and Supplementary Table S2), hence showing the effectiveness and relevance of our ranking approach. The gene with the highest ranking in humans was *POU5F1*, found in 12 of the individual human stemness signatures (Supplementary Table S2).

*Phc1* gene obtained the highest-ranking score of 13 in the mouse ISS (Supplementary Table S3). *Phc1* codes for a protein of the PolyComb repressive complex 1 (PRC1) required to maintain the transcriptionally repressive state of many genes via chromatin remodelling and histone modification [47]. It has been involved with DNA repair in yeast [48], as well as in the maintenance of the proliferation capability and self-renewal ability of hematopoietic stem cells [49].

For the mouse ISS, we noted an absence of key pluripotent marker genes such as *Pou5f1* (Oct4 protein), *Sox2*, and *Nanog* among the top-scoring genes (Figure 1B). Those three genes were present in the mouse ISS, however with lower scores (scores 6, 8, and 7, respectively) due to the lower percentage of stemness signatures derived from pluripotent cells for mice compared to humans. To alleviate potential biases due to this difference, we additionally calculated distinct scores for pluripotent and multipotent stemness signatures (Supplementary Tables S2 and S3). Visualization of the presence in stemness signatures showed a considerable reordering among the genes with the highest pluripotency and multipotency scores (Supplementary Figures S4 and S5). The top human genes based on the multipotency scores were all included in the hemopoietic stem cell (HSC) signatures, with a subset such as *DNMT3B*, *MYCN*, and *PROM1* also included in signatures for pluripotent stem cells (Supplementary Figure S4B). For mice, ordering based on the pluripotency scores led to the appearance of *Pou5f1* (Oct4 protein), *Sox2,* and *Nanog* among the top-scoring genes (Supplementary Figure S4C), while other genes such as *Phc1* and *Trim28* retain a relatively high score.

In general, genes of the ISS were included in both pluri- and multipotent signatures, indicating shared biological features between pluri- and multipotency. However, a subset of genes was exclusively associated with either pluri- or multipotent stemness signatures. For instance, if we require that genes should be included in at least 30% of the stemness signatures of one potency class, and not in any of another potency class, we would obtain 61 human pluripotency- and 100 multipotency-specific genes, as well as 34 murine pluripotency- and 175 multipotency-specific genes. The top-scoring genes are presented in Supplementary Figure S5, and include, for instance, Frizzled Class Receptor 2 (*Fzd2*), which is a receptor in the Wnt pathway, among the multipotency-specific murine genes.

Notably, 17 orthologous genes were shared between mouse and human ISSs (Supplementary Table S6), forming an evolutionarily conserved core of stemness. For this set of conserved genes, we calculated an overall score for each gene as the sum of the scores for that gene in human and mouse ISSs (Supplementary Table S6). The largest scores were obtained for *Pou5f1*, *Sox2*, *Mycn*, and *Msh2*. Among these top genes, *Msh2* appears to be the least studied in the context of stem cell biology. *Msh2* is part of the post-replicative DNA mismatch repair system and is frequently mutated in hereditary nonpolyposis colon cancer [47].

Both human and mouse ISSs genes showed significant enrichment in all groups tested, namely, biological processes, molecular functions, cellular components, and Reactome pathways (Supplementary Material: *Functional Analysis of integrated stemness signatures*, and Supplementary Figure S6), further validating the association of ISSs and stemness. Furthermore, we obtained distinct functional profiles when we compared genes that were highly ranked based on pluripotency and multipotency scores, or were exclusively associated with pluripotent or multipotent stemness signatures based on the previous definition (Supplementary Figure S7). Comparing the top 200 human genes for pluripotency and multipotency, both groups shared a significant enrichment of the GO term *stem cell population maintenance*, while *somatic stem cell population maintenance* was more significantly enriched among multipotency genes and *DNA replication* was only significantly enriched (adj. *p*-value < 0.05) among pluripotent genes (Supplementary Figure S7A). For murine genes exclusively associated with pluripotency, we found that genes in the GO term response to *leukemia inhibitory factor* (LIF) were significantly overrepresented, reflecting the typical supplementation of LIF to the culture medium of murine ESCs (Supplementary Figure S7D). In contrast, genes associated with *epithelial cell proliferation* and *regulation of hemopoiesis* were overrepresented among exclusively multipotency genes.

### 3.2. Integrative Stemness Signature Reveals Genes Whose Function Has Not Been Linked Yet to Stem Cells

Inspection of ISSs reveals many genes that have already been linked in previous studies to the properties of stem cells. Nevertheless, these integrated signatures might also contain genes that, while consistently associated with many stem cell signatures (and thus high ranking in the ISSs), have been rarely, or not at all, the focus of dedicated experimental stem cell studies.

To identify genes that tended to be included in stemness signatures but whose function has not been linked yet to stem cells, we conducted a text-mining approach. We derived for each gene of human and mouse ISSs the number of associated publications in PubMed that include the term “*stem cell(s)*” in the full-text version of the articles or only in their titles or abstracts. This number should provide a measure for conducted research on a specific gene in stem cell biology, and was subsequently plotted against the previously calculated scores for occurrences in stemness signatures.

**Figure 2:**
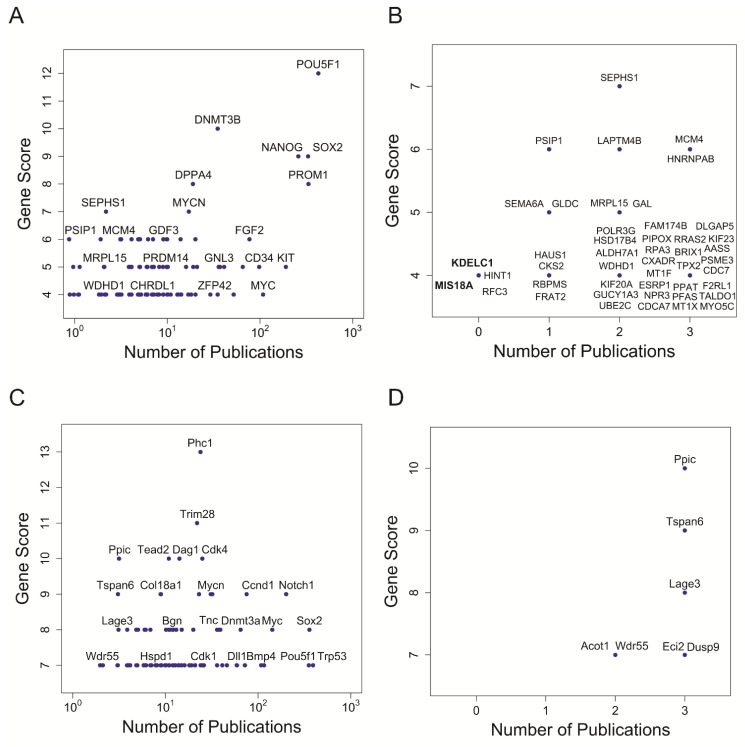
Identification of stemness genes overlooked in individual studies. Number of occurrences in individual stemness signatures versus number of stem cell-related publications for each gene in human ISS (**A**,**B**); and in mouse ISS (**C**,**D**). Number of publications is defined by the number of all articles containing the gene name together with the term stem cell(s) in the title or abstract in PubMed. (**A**,**C**) show gene names with at least one stem cell-related publication. Note, not all genes are labelled and the number of publications is on a logarithmic scale (log_10_). (**B**,**D**) show genes referenced in only up to 3 stem cell-related publications. Bold font highlights gene names that had never been referred to together with the term stem cell in the full text of any article in PubMed.

For most genes of the human ISS, we found co-citation with the term *stem cell(s)* in PubMed (Figure 2A). Notably, ISS genes with the highest score have a substantial number of publications, demonstrating that they have been well studied in the context of stem cell research. For example, the high-scoring master regulators of pluripotency (*POU5F1*, *SOX2*, *NANOG*) were among the genes with the most stem cell-associated publications. However, there are some notable divergences. For instance, *SEPHS1*, which is involved in the selenium metabolism [47] with a relatively high score of 7, was only associated with stem cells in two publications [50,51] at the time of analysis (Figure 2B). Strikingly, four genes (*RFC3*, *MIS18A*, *HINT1*, and *KDELC1*) have never been named together with the term *stem cell(s)* in the title or abstract of PubMed articles at the time of the conducted literature mining (Figure 2B), although they were found in 4 stemness signatures (Supplementary Table S2). *MIS18A* and *KDELC1* are particularly attractive candidates for further study since they never co-occurred with the term *stem cell(s)* in any part (not even the main body) of a PubMed article. *MIS18A* codes for a protein essential for the recruitment of the centromere protein A (CENPA) to centromeres, hence being pivotal for normal chromosome segregation during mitosis [52]. Mouse phenotypes associated with *MIS18A* are *Embryo* and *Mortality/Aging* [53]. *KDELC1* codes for a protein found in the lumen of the endoplasmic reticulum (ER) containing a motif (KDEL) that prevents its secretion to the outside [47]. Very little is known about the function of *KDELC1*, although it has been associated with the molecular function of glucosyltransferase, and with the biological processes of carbohydrate and lipid metabolism [54]. We propose that these two genes could be novel players in the context of stem cell biology, and hence good candidates for further experimental studies. Notably, *MIS18A* was recently independently suggested as a biomarker for leukaemia stem cells based on bioinformatics analysis of the Cancer Genome Atlas [55]. For the mouse ISS, every gene occurred in at least one PubMed reference together with the term stem cell(s) in the title or abstract (Figure 2C,D).

### 3.3. Network Analysis of Integrated Stemness Signatures

Many cellular functions are based on the interactions of proteins. Thus, we expect that proteins encoded by genes frequently found in stemness signatures interact with one another to perform processes that are important for stem cells. Therefore, we built protein interaction networks for enlarged ISSs containing the genes with minimum scores of 3 and 6 respectively, for the human and mouse ranked lists.

**Figure 3:**
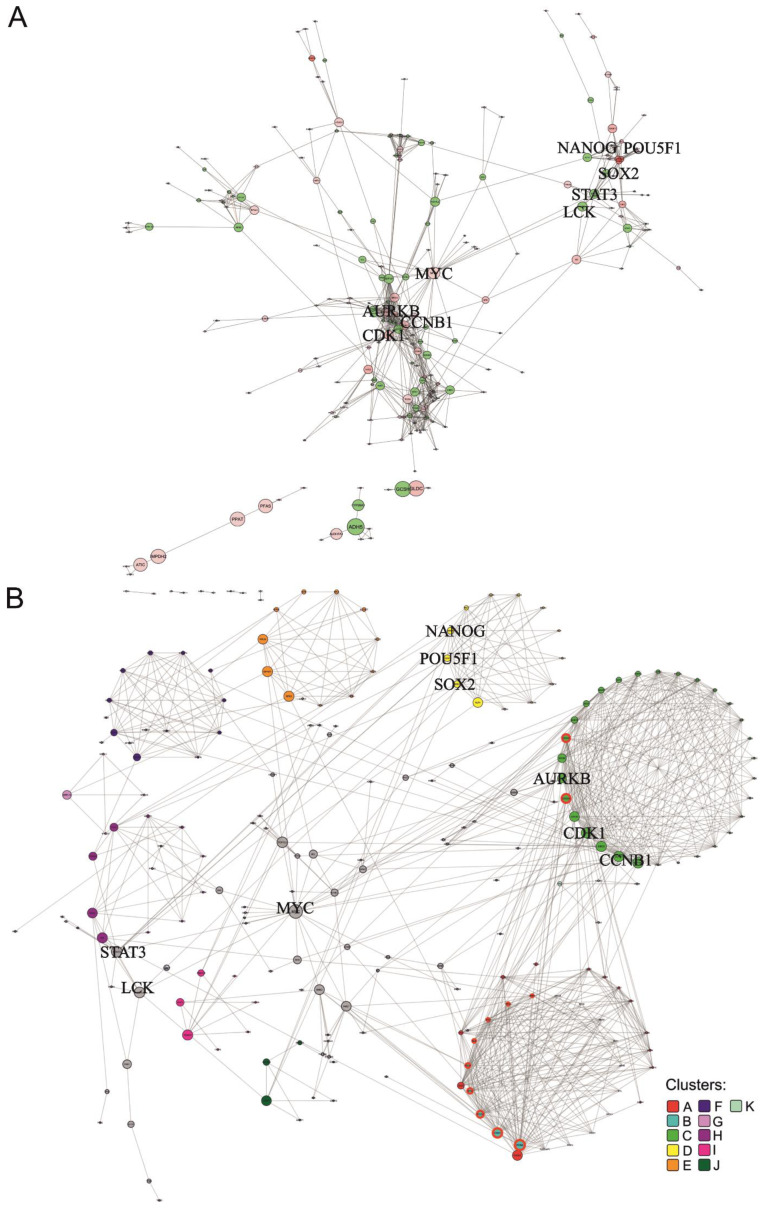
Protein network of human ranked list. Interactions of proteins corresponding to genes with a minimum score of 3 in the human ranked list are shown. Bold font highlights nodes referred to in the main text. (**A**) Overall network visualized with the edge-weighted spring embedded layout. Red nodes represent genes that belong to the human ISS (with score ≥ 4). Edge thickness reflects the interaction confidence score, whereas node size and colour opacity are proportional to node betweenness centrality and the stemness score of the gene, respectively. (**B**) Significantly interacting network clusters (*p*-value < 5 × 10^−2^). Nodes of each cluster are placed in circles according to their betweenness centrality. Nodes of a cluster share the same colour (see legend) and node size is proportional to node betweenness centrality. Nodes without interactions were excluded. Nodes that did not interact with the main network were excluded before the clustering analysis.

The human network comprised 232 nodes and 822 edges in total (Figure 3A). On average, each node is linked to 3.5 other nodes. The nodes with the highest degree centralities (i.e., number of direct connections) are *CDK1*, *CCNB1,* and *AURKB* with 34, 30, and 27 interactions, respectively (Supplementary Table S7). The first two (cyclin-dependent kinase, and cyclin B1) are well-known regulators of the cell cycle. *AURKB* is part of the aurora kinase subfamily of serine/threonine kinases participating in the regulation of alignment and segregation of chromosomes during mitosis and meiosis through association with microtubules [47].

Visual inspection suggested that several interconnected clusters exist and pointed to proteins that are important for the structural cohesiveness of the network. To further explore the community structure of the network, a graph-based clustering approach was carried out and revealed 11 significant clusters (*p*-value < 5 × 10^−2^) (Figure 3B and Supplementary Figure S8). These clusters are not mutually exclusive. For example, some nodes (genes) of cluster A are also nodes of cluster C (light green nodes with a red border in Figure 3B), while cluster B is a sub-cluster of cluster A (light blue nodes with a red border). We observe that the clusters with higher ranking significance are clusters A, C, and D (‘*p*-value rank’ column Supplementary Table S4). Functional enrichment analysis based on KEGG gene annotations showed that cluster A is enriched in *DNA synthesis* and *cell cycle checkpoints*, cluster B is mainly related to *DNA repair* and *telomere synthesis and extension,* while genes in cluster C tend to be associated with mitotic phases (Supplementary Table S8). Cluster D includes the main transcription regulators *POU5F1*, *SOX2,* and *NANOG*, explaining its enrichment in the *Transcriptional regulation of pluripotent stem cells*. Besides ranking third for cluster significance, cluster D is also the one showing the highest average stemness score (‘Average stemness score rank’ column Supplementary Table S4). The cluster with the second-highest average stemness score is cluster I (Supplementary Table S4), which is associated with *Epigenetic regulation of gene expression* and *RNA Polymerase I Transcription* (Supplementary Table S8).

The calculation of betweenness centrality, defined by the number of times each node lies in the shortest path connecting two other nodes divided by the total number of shortest paths, was used for the stringent identification of proteins that are most important for the integrity of the network. We found that *MYC* has the largest betweenness centrality (Supplementary Table S7) and connects, directly and indirectly, several clusters (clusters A–F and H, Figure 3B). *MYC* is a proto-oncogene that forms a dimer with the MAX transcription factor, regulating the transcription of genes related to cell cycle progression, apoptosis, and cellular transformation. It is also one of the Yamanaka factors for induced pluripotency [47]. *MYC* is among the nodes with the highest degree of centrality values, describing the number of nodes with which a node is directly linked. Together with *MYC*, *LCK,* and *STAT3* are the nodes of the main network with the highest betweenness centralities (Supplementary Table S7). *LCK* is a proto-oncogene and an important signalling molecule in the maturation of developing T-cells [56], contributing to the hematopoietic system and immune system phenotypes in mice [53], whereas *STAT3* is a member of the STAT family of proteins, which mediate cell growth and apoptosis among other processes in response to cytokines and growth factors [57]. Although there are other proteins with higher betweenness centrality, those were found in small isolated clusters and their betweenness centrality values do not reflect node relevance to the overall network (greyed-out proteins in Supplementary Table S7). The proteins corresponding to genes with larger stemness scores, OCT4 (encoded by *POU5F1* gene), NANOG, and SOX2, present medium node degree and betweenness centralities (Supplementary Table S7).

**Figure 4:**
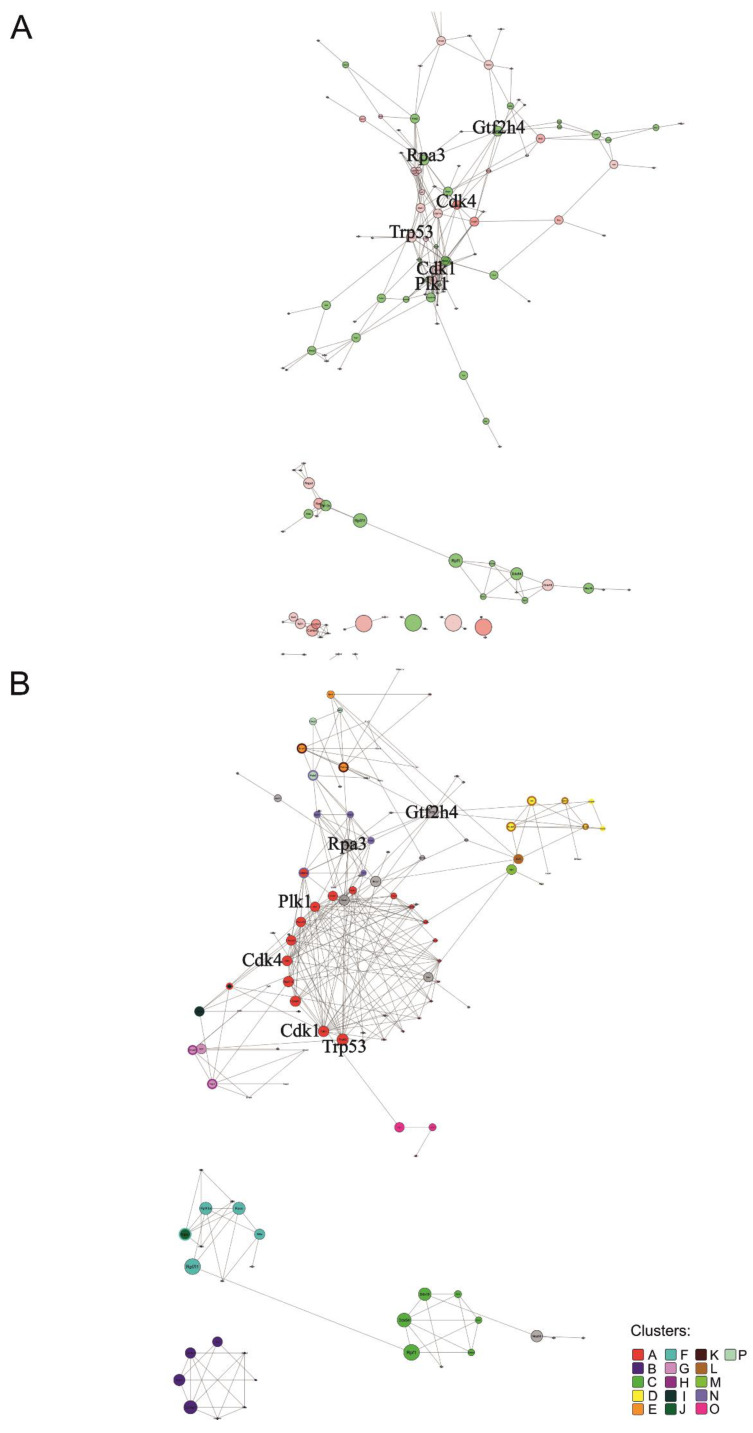
Protein network of mouse ranked list. Interactions of proteins corresponding to genes with a minimum score of 6 in the mouse ranked list are shown. Bold font highlights nodes referred to in the main text. (**A**) Overall network visualized with the edge-weighted spring embedded layout. Red nodes represent genes that belong to the mouse ISS (with score ≥ 7). Edge thickness reflects the interaction confidence score, whereas node size and colour opacity are proportional to node betweenness centrality and the stemness score of the gene, respectively. (**B**) Significantly interacting network clusters (*p*-value < 5 × 10^−2^). Nodes of each cluster are placed in circles according to their betweenness centrality. Nodes of a cluster share the same colour (see legend) and node size is proportional to node betweenness centrality. Nodes without interactions were excluded. Nodes that did not interact with the main network and were part of a smaller network (with less than four nodes) were excluded before the clustering analysis.

The mouse network contained 134 nodes and 280 edges in total (Figure 4A), where each node interacted on average with 2.1 other nodes. *Cdk1* and *Plk1* are the nodes that present the highest degree centralities, 21 and 16, respectively (Supplementary Table S9). *Plk1* is a protein kinase regulating the cell cycle, cytokinesis, and DNA damage response, while *Cdk1* is part of the cell cycle protein complex [47]. Among nodes with the largest stemness score, the one with the highest node degree and betweenness centrality is *Cdk4* (Supplementary Table S9), which is a kinase with an important role in the cell cycle [47]. We identified 16 significant clusters (*p*-value < 5 × 10^−2^) in the mouse network (Figure 4B and Supplementary Figure S9). Clusters with higher cluster significance are A and B (‘*p*-value rank’ column Supplementary Table S5). Cluster A is enriched in proteins associated with *cell cycle phases* and *mitotic checkpoints*. Surprisingly, cluster B is related to *extracellular matrix pathways* and *cell interaction* (Supplementary Table S10). Clusters ranking higher for average stemness score are B and J (‘Average stemness score rank’ column Supplementary Table S5). Cluster J is related to gene *transcription and post-transcriptional processing* (Supplementary Table S10). *Rap3* and *Gtf2h4* code for proteins connecting directly or indirectly most clusters of the network (clusters A, D, E, G, H and clusters J-P, Figure 4B) and are together with *Trp53* the nodes of the main network with the highest betweenness centralities. Those 3 proteins are also among the nodes demonstrating high degrees of centrality (Supplementary Table S9). *Rap3* is reported as having a role in the regulation of plasma triglyceride levels. It is a component of high-density lipoprotein and is very similar to a rat protein that is upregulated in response to liver injury [58]. In mice, it is associated with homeostasis and metabolism phenotype [59]. *Gtf2h4* is a component of a transcription factor involved in nucleotide excision repair of DNA and, when complexed with CAK, in transcription, while Trp53 is a tumour suppressor transcription factor [47].

### 3.4. Limitations of the Integrative Analysis of Stem Cell Signatures

In contrast to conventional meta-analysis, our analysis was not based on pooling effect sizes such as differential expression observed in individual studies, but on pooling the final dichotomous results, i.e., the presence or absence of genes in the ISSs. While this approach might have led to a loss of statistical power (e.g., detecting genes with small expression changes), it facilitated the integration across different underlying methodologies (i.e., transcriptomic analysis, knock-down screens, literature curation and computational approaches).

Importantly, the absence of a gene in the ISSs does not necessarily signify that it does not play a role in stem cells. This can be illustrated with members of the Polycomb group (PcG) genes, whose role for stem cells has been intensively studied [60]. Although *Phc1* was the gene with the highest score in the murine ISS, confirming its experimentally established role in stem cell maintenance [61], many PcG genes have either low scores or were absent in the ISSs (Supplementary Figures S10A and S11A). Although such absence appears unexpected, it is a consequence of methodological limitations of the approaches defining stemness signatures and the propensities of affected genes. For instance, expression-based stemness signatures use over-expression in stem cells to define stemness-associated genes and thus miss genes that are important for stem cell biology but do not show higher transcript levels in stem cells. Expression data from the StemMapper database [26], which merges expression data for stem cells and various cell lineages, indicate that this is also the case for PcG genes (Supplementary Figures S10B and S11B). Compared to *Nanog*, downregulation of gene expression in differentiated cells is less prominent or not apparent at all for PcG genes. Furthermore, RNAi-based stemness signatures depend on observable effects (measured by a chosen assay) of single gene knockdowns. If redundancies between stemness genes exist, knockdown or knockouts of single genes might not be sufficient to reveal their role. For PcG genes, for example, recent experiments demonstrated widespread functional redundancies, as single PcG gene knockouts did not result in changes in *Pou5f1* and *Sox2* expression in murine ESCs [62]. This was also reflected in the RNAi-based stemness signatures, which did not include any PcG genes apart from one exception (Supplementary Figures S10A and S11A).

Due to these limitations in the underlying methodologies, an absence of genes from the ISSs should not be taken as an indication that they do not play a role in stem cell biology. Rather, our compendium of stemness signatures and the derived ISSs can point to genes whose functional role in stem cells has been less studied or not investigated at all. Thus, it may serve as a rational evidence basis to broaden stem cell research [63].

## 4. Conclusions

In conclusion, the application of genome-wide profiling techniques greatly facilitated the detection of stemness genes. While many studies generated gene signatures for various types of stem cells, caution in their interpretation is warranted due to the biological heterogeneity of stem cell populations, as well as the technical variability of profiling platforms and protocols. Indeed, earlier comparisons of stemness signatures yielded limited overlap even for the same microarray platform. Such study-specific effects can be mitigated by comparing a greater number of stemness signatures obtained from several different experimental approaches for different stem cell types. With this meta-analysis approach, to the best of our knowledge the largest to date, we were able to detect trends based on the overlap of individual stemness signatures for humans and mice. Despite the lack of genes common to all stemness signatures, our study revealed significant overlap between most individual signatures. Therefore, it was possible to define an ISS comprised of genes most frequently found among individual studies. With this signature, we expect to enhance confidence regarding the genes associated with stemness, and help to exclude false positives that can compromise individual studies. The relevance of our approach and results is supported by the fact that mouse and human signatures were functionally enriched in biological processes, molecular functions, and cellular components, as well as pathways related to stem cell properties. Importantly, we could pinpoint stemness genes that occurred frequently in stemness signatures but have eluded dedicated studies in stem cell biology so far. Such under-researched genes can provide prime targets for future investigations elucidating the molecular basis of stemness.

## Data Availability

All data supporting the reported results is openly available in the Supplementary Tables S1–S10.

## Supplementary Materials

The supporting information can be downloaded at: https://www.mdpi.com/article/10.3390/genes14030745/s1. Figure S1: Significance of overlap of genes between individual stemness signatures; Figure S2: Distribution and significance of stemness scores for human genes; Figure S3: Distribution and significance of stemness scores for mouse genes; Figure S4: Association of human and mouse genes with stemness signatures; Figure S5: Human and mouse genes specific to pluripotent or multipotent stemness signatures; Figure S6: Functional characterization of Integrated Stemness Signatures; Figure S7: GO enrichment analysis for genes associated with pluripotency or multipotency stemness signatures; Figure S8: Complementary figure to Figure 3; Figure S9: Complementary figure to Figure 4; Figure S10: Stemness association and expression of human Polycomb group (PcG) genes; Figure S11: Stemness association and expression of murine PcG genes; Table S1: Description of individual stemness signatures; Table S2: Human stemness genes ranked list; Table S3: Mouse stemness genes ranked list; Table S4: Significant clusters of human ISS network; Table S5: Significant clusters of mouse ISS network; Table S6: Evolutionary conserved stemness genes; Table S7: Node betweeness and degree centrality for network of human ISS; Table S8: Reactome pathways overrepresented in significant clusters of human ISS network; Table S9: Node betweenness and degree centrality for mouse ISS network; Table S10: Reactome pathways overrepresented in significant clusters of mouse ISS network [6,7,8,11,12,13,14,15,16,17,18,19,20,21,22,23,24,25,27].
