# Supplementary material for: Integration of Stemness Gene Signatures Reveals Core Functional Modules of Stem Cells and Potential Novel Stemness Genes
Source: Genes (Basel). 2023 Mar 18;14(3):745. doi: 10.3390/genes14030745 (PMC10048104; doi:10.3390/genes14030745)
Supplement: Supplementary file 1 [file genes-14-00745-s001.zip › Supplementary_Materials Figures.pdf]

Supplementary Materials

Influence on stem cell origin and methodology on individual stemness signatures

Individual human or mouse stemness signatures were clustered based on the significance of the pairwise overlap of their genes. In total, 119 of the 210 pairwise comparisons for human, and 148 of the 210 comparisons for mouse signatures led to the detection of significant overlap, with adjusted p-value $< 5 \cdot 10^{-2}$, i.e. $\log_{10}$(adjusted p-value) < -3.1 in Figure S1.

For human stemness signatures, two main clusters were detected (Figure S1A). The first cluster (C_h1) is mainly composed of pluripotent stemness signatures derived from gene expression profiling (*Hs_ESC_Bhattacharya*[1], *Hs_ESC_Assou*[2], *Hs_ESC_Wong*[3], *and Hs_SC_Palmer*[4]). It also includes *PluriNet*[5] data which is a computationally derived protein network shared by different types of pluripotent cells (embryonic stem cells, embryonal carcinomas, and induced pluripotent cells). Interestingly, gene sets for cancer cells (*Hs_EC_Skotheim*[6], *Hs_SC_Shats*[7], and *Hs_ESC_EC_Sperger*[8]) are also part of this cluster, implying similarity of gene expression profiles between pluripotent and cancer cells.

The second main cluster (C_h2) is more heterogeneous. There is a sub-cluster composed of gene sets for hematopoietic stem cells (HSCs): *Hs_HSC_Toren*[9], *Hs_HSC_Huang*[10], and *Hs_HSC_Novershtern*[11]. In a different sub-cluster, the grouping *Hs_iPSC_Shats*[7], *Hs_ESC_Skottman*[12], and *Hs_ESC_Sato*[13] might reflect the shared pluripotent nature of ESC and iPSC stem cells. In contrast, other ESCs gene sets such as *Hs_ESC_Chia*[14] do not cluster together with this group, suggesting that the different methods (microarrays versus RNAi screens) and even the type of microarray platform (Affymetrix versus customizable spotted microarrays) might influence the results of the clustering (Figure S1A and Supplementary Table 1). Another sub-cluster is formed by gene sets based on literature curation or text mining: *Reactome*, *KEGG,* and *Genecards*.

For the mouse stemness signatures, we also obtained two main clusters (Figure S1B). The first cluster (C_m1), shows the impact of the methodology factor, namely the type of microarray platform used for the experiment. While it can be expected that *Mm_ESC_Ramalho*[15] is paired with *Mm_ESC_Fortunel*[16] since they share the same stem cell type, the inclusion of *Mm_NSC_Ramalho*[15] in the same sub-cluster points to the influence of the platform on the detected overlap. Similarly, *Mm_NSC_Fortunel*[16] and *Mm_RPC_Fortunel*[16] clusters with *Mm_HSC_Ramalho*[15], while *Mm_NSC_Ivanova*[17] clusters with *Mm_HSC_Ivanova*[17].

The second cluster comprises ESC gene sets and a gene set for spermatogonial stem cells (SSCs), the *Mm_SSC_Kokkinaki*[18]. This is following previously published results[19] indicating that SSCs acquire pluripotent stem cell properties when cultured *in vitro*. This finding cannot be explained as the result of the

technological platform used, since most ESC sets of the cluster were obtained from iRNA screens, while the *Mm_SSC_Kokkinaki* set is the product of an Affymetrix microarray experiment (Figure S1B and Supplementary Table 1).

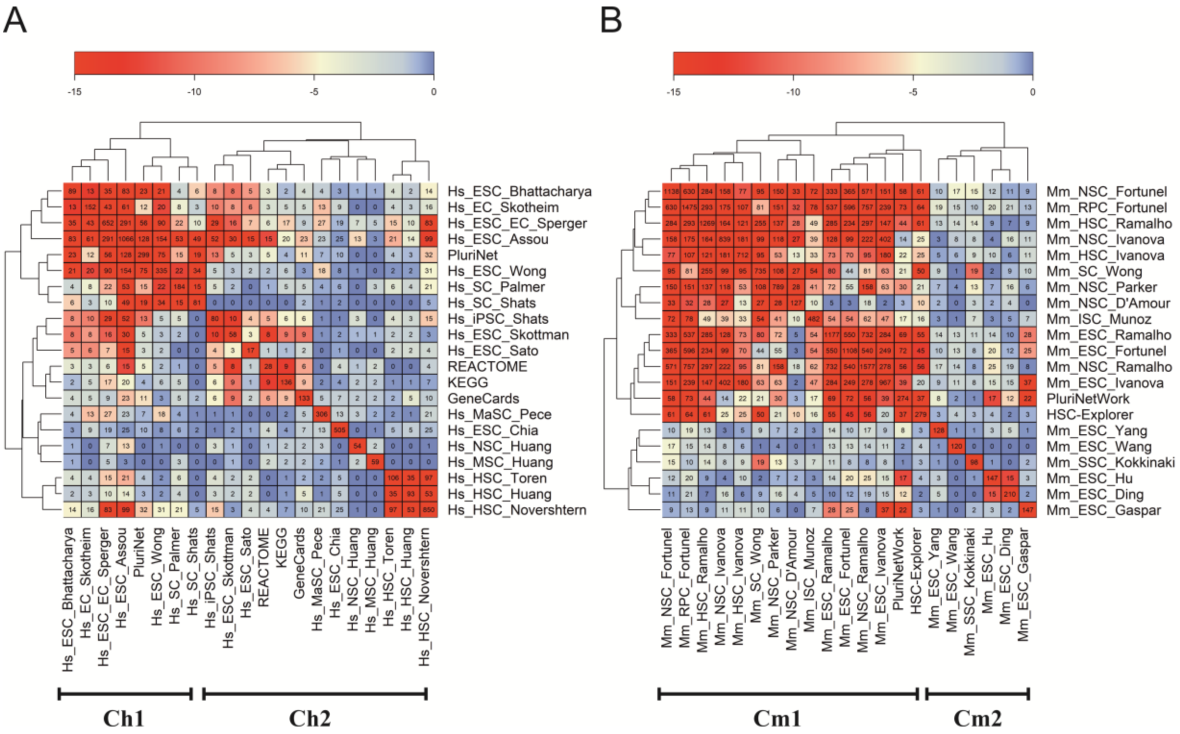

Figure S1 – Significance of overlap of genes between individual stemness signatures

Significance of overlap of genes, shown as $\log_{10}$(adjusted p-value), between individual stemness signatures for human **(A)** and for mouse **(B)**. **Ch1** and **Ch2** are clusters for human, while **Cm1** and **Cm2** are clusters for mouse signatures. Row and Column dendrograms are based on the Euclidean distance between cluster objects and are derived by complete linkage as an agglomeration method. Number of genes common to a pair of stemness signatures is shown inside each square.

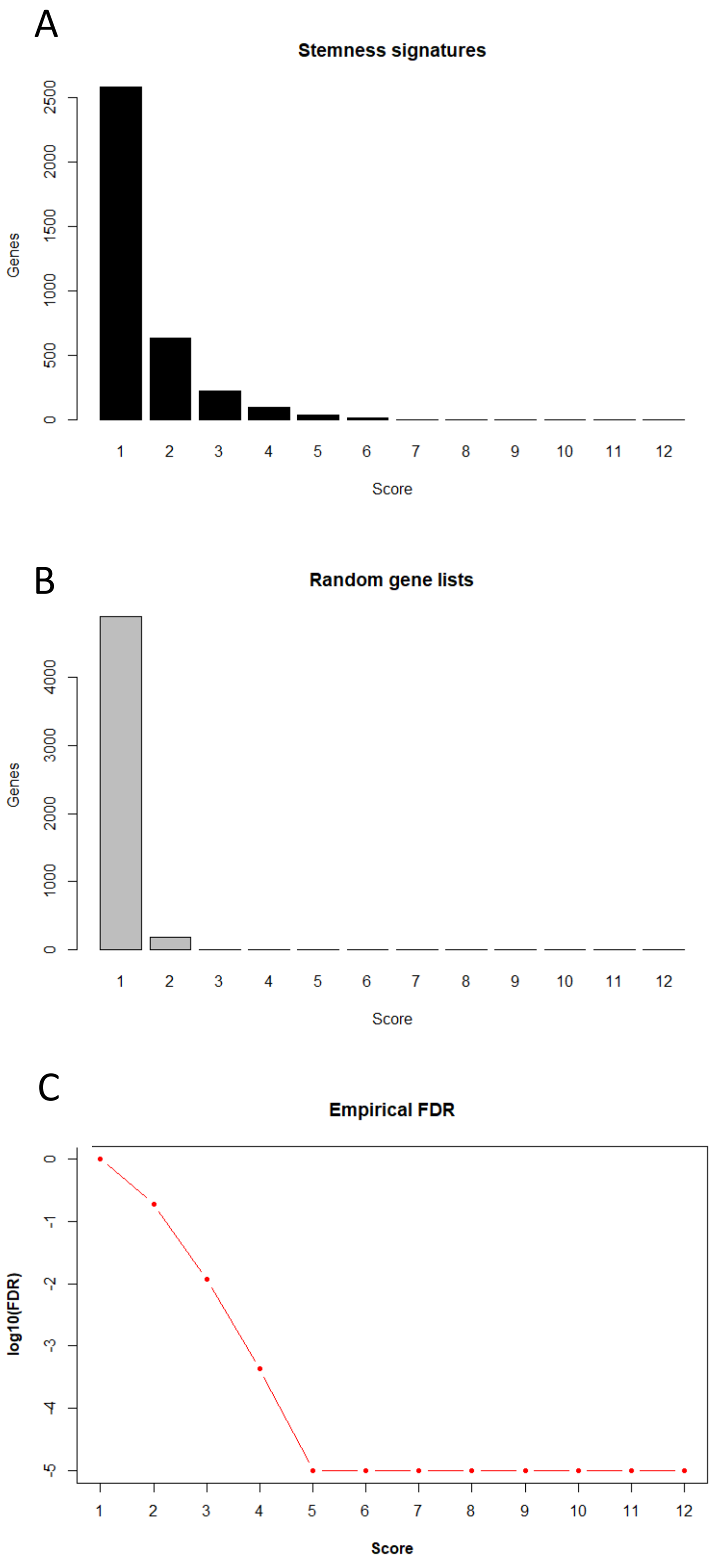

Figure S2 – Distribution and significance of stemness scores for human genes. Observed scores for human stemness signatures (**A**), average scores for randomly drawn gene lists of the same size (**B**), empirical FDR based on comparison of observed and expected distribution of scores (**C**). A minimum FDR of $1 \cdot 10^{-5}$ (i.e. the inverse of the number of random draws) was set.

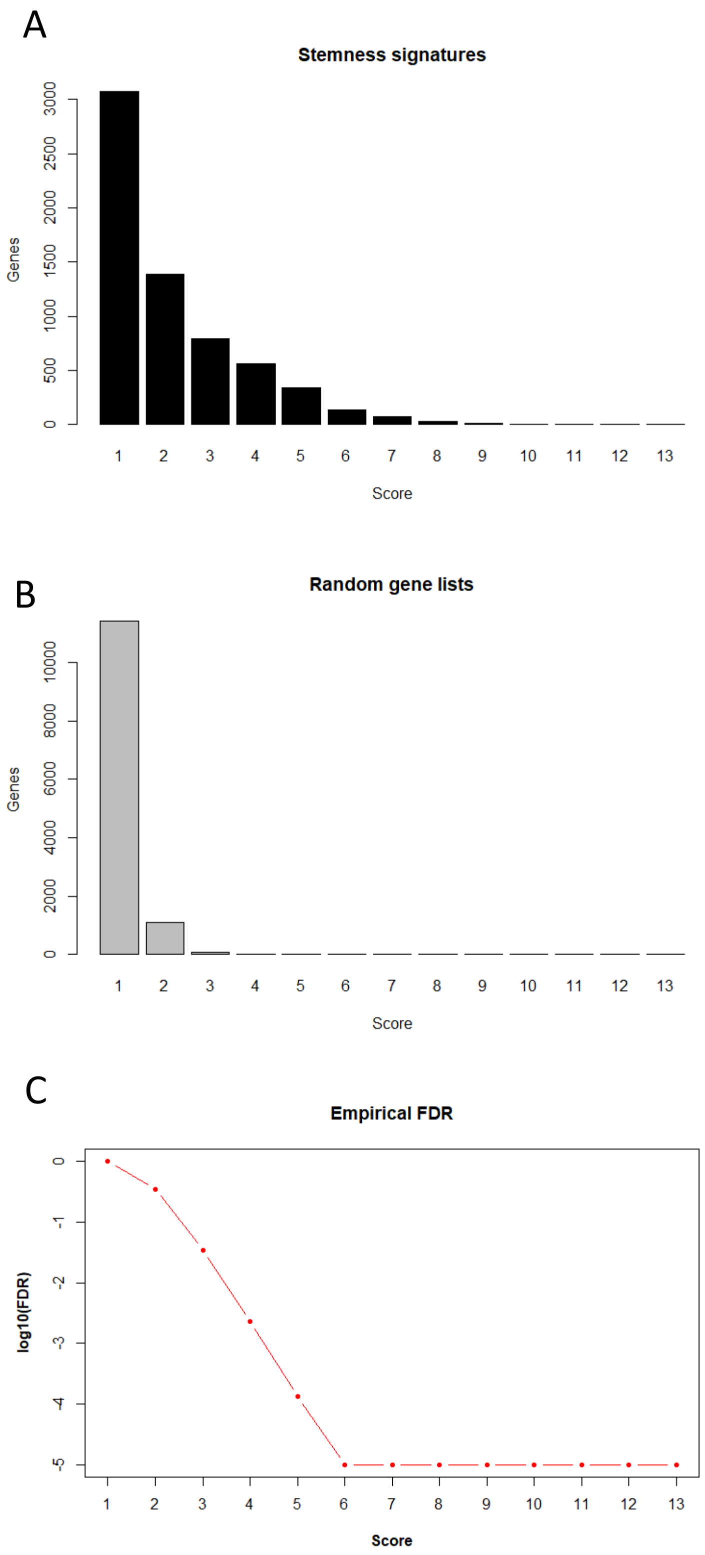

Figure S3 – Distribution and significance of stemness scores for mouse genes. Observed scores for mouse stemness signatures (**A**), average scores for randomly drawn gene lists of the same size (**B**), empirical FDR based on comparison of observed and expected distribution of scores (**C**). A minimum FDR of $1 \cdot 10^{-5}$ (i.e. the inverse of the number of random draws) was set.

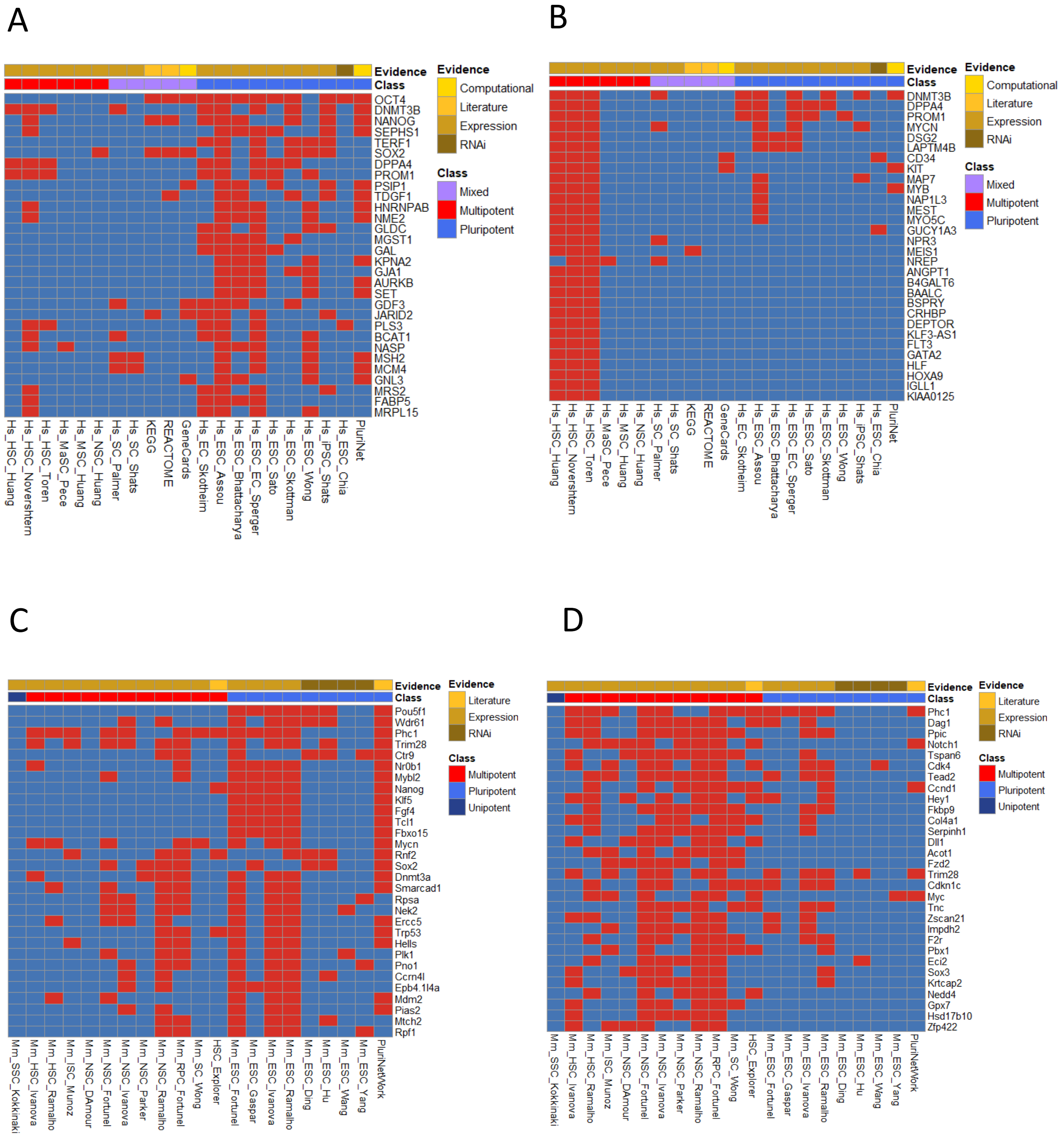

Figure S4 – Association of human and mouse genes with stemness signatures. In the checkerboards, red colour indicates the inclusion of genes (row) in stemness signature (column). The evidence for the stemness signatures (Computational, Literature, Expression, RNAi) and the type of stem cell based on their potency (Pluripotent, Multipotent, Mixed) is annotated on top of the checkerboards. **(A)** The 30 human genes with the highest pluripotency score. **(B)** The 30 human genes with the highest multipotency scores. (**C**) The 30 mouse genes with the highest pluripotency score. (**D**) The 30 mouse genes with highest multipotency score.

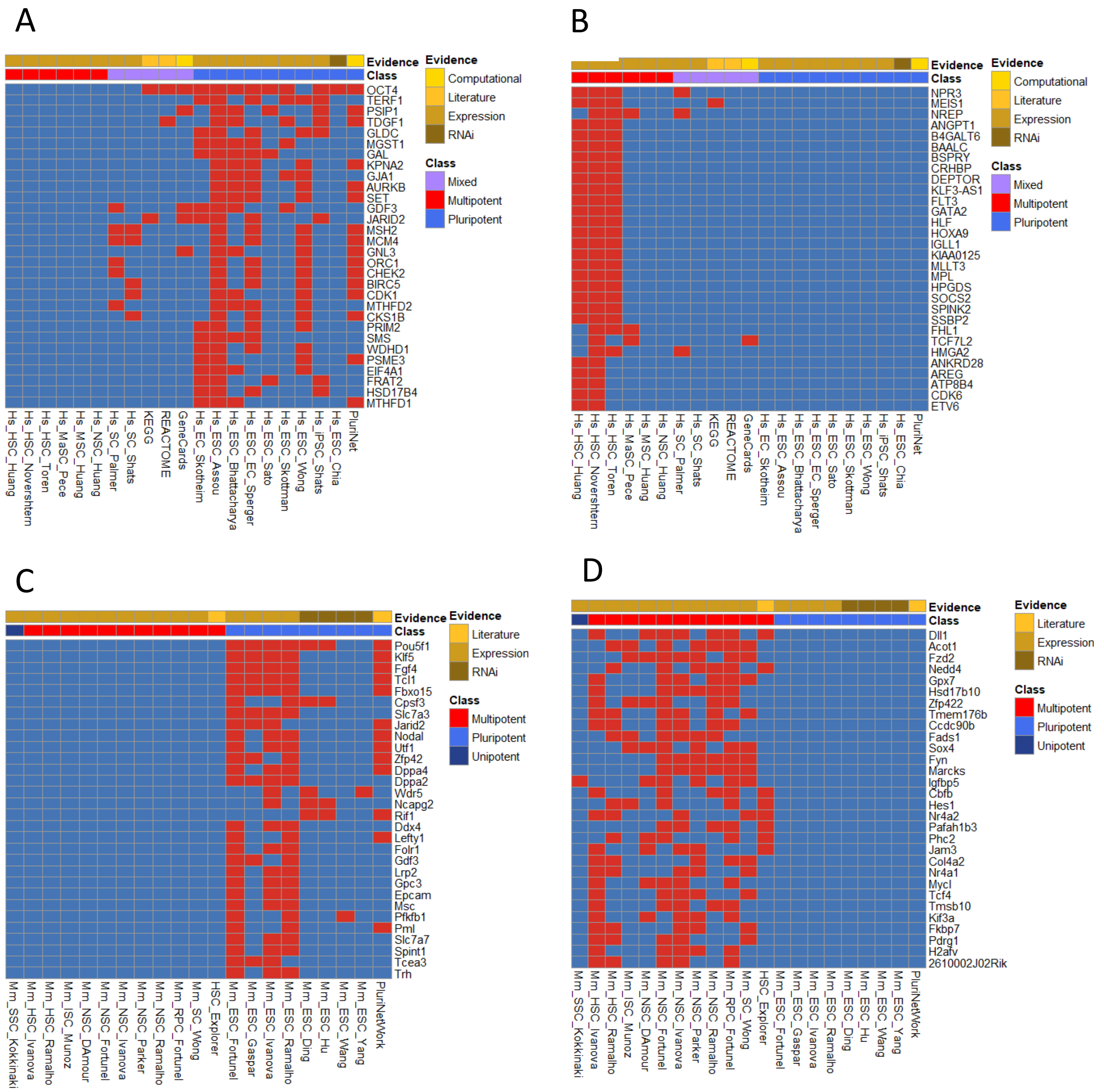

Figure S5 – Human and mouse genes specific to pluripotent or multipotent stemness signatures. In the checkerboards, red colour indicates the inclusion of genes (row) in stemness signature (column). The evidence for the stemness signatures (Computational, Literature, Expression, RNAi) and the type of stem cell based on their potency (Pluripotent, Multipotent, Mixed) is annotated on top of the checkerboards. **(A)** The 30 human genes, which have the highest pluripotency score but do not appear in stemness signatures for multipotent stem cells. **(B)** The 30 human genes, which have the highest multipotency score but do not appear in stemness signatures for pluripotent stem cells**.** **(C)** The 30 murine genes, which have the highest pluripotency score but do not appear in stemness signatures for multipotent stem cells. **(D)** The 30 murine genes, which have the highest multipotency score but do not appear in stemness signatures for pluripotent stem cells.

Functional Analysis of integrated stemness signatures

Human and mouse ISSs genes showed significant enrichment in biological processes related to the characteristic properties of stem cells. For example, processes related to mitosis, cell cycle, and DNA replication underlying self-renewal (in bold in Figure S4A). Molecular functions related to nucleotide and ATP binding are strongly overrepresented, suggesting the participation of ISS genes in DNA replication and transcription, as well as in metabolism (in bold in Figure S4B). These signatures are also associated with all types of cellular components, and one of the most enriched is nucleus. Furthermore, following the enrichment for the DNA replication process, ISS genes display an enrichment for the MCM complex, a hexameric protein complex required for the initiation and regulation of DNA replication (in bold in Figure S4C). Enrichment analysis based on Reactome pathways showed DNA damage/replication checkpoints and cell cycle phase transitions/regulation which are among the most enriched terms in both human and mouse (in bold in Figure S4D).

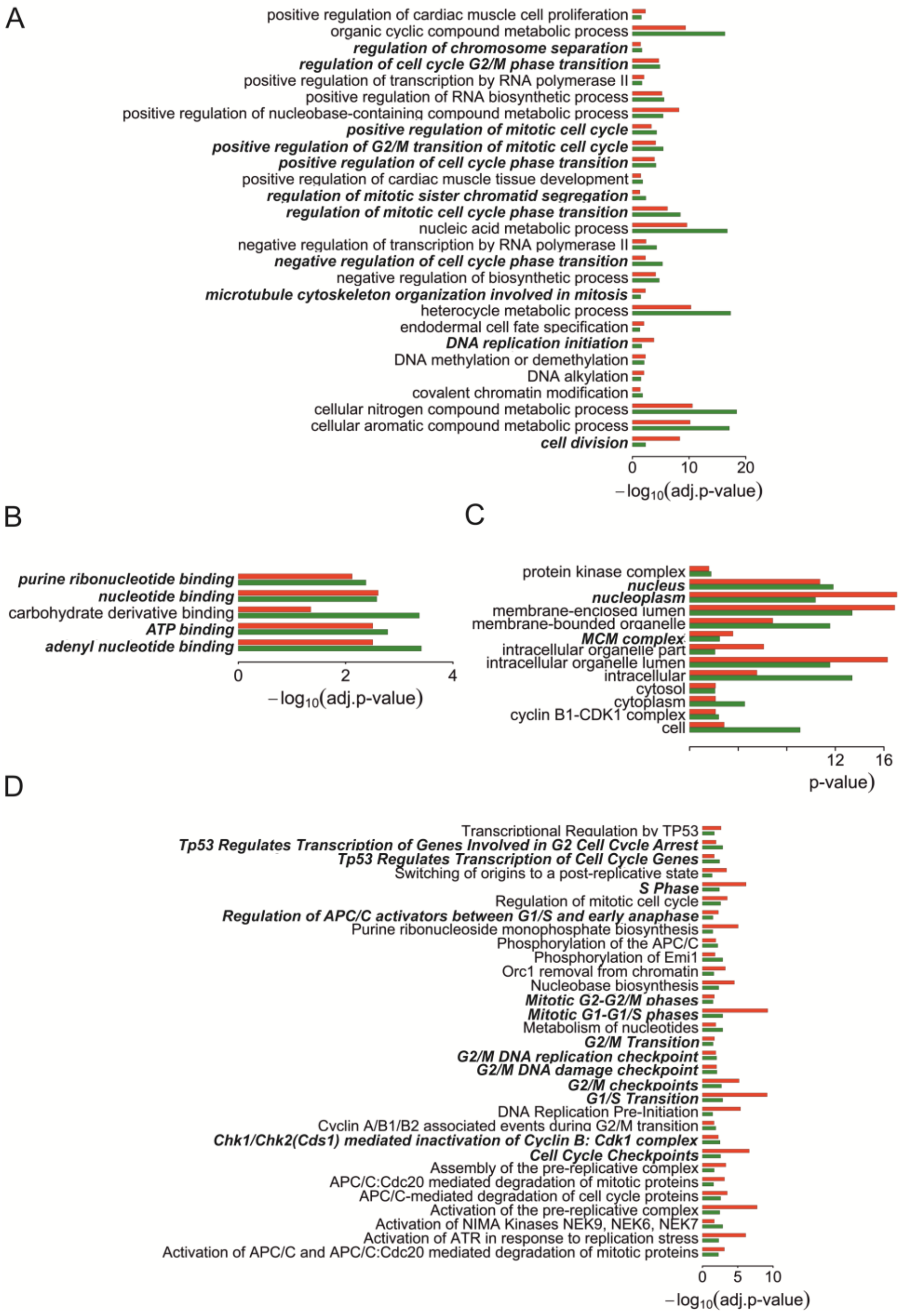

Figure S6 – Functional characterization of *Integrated Stemness Signatures*

Biological Processes (**A**), Molecular Functions **(B)**, Cellular Components **(C)** and Reactome Pathways **(D)** overrepresented (adj. p-value < $5 \cdot 10^{-2}$) on both human and mouse *ISSs*. Red (top) and green (bottom) bars represent human and mouse respectively. Bold font highlights functional categories referred to in the main text: processes related with mitotic cell cycle and DNA replication; molecular functions related with nucleotide and ATP binding; cellular components related with nucleus and MCM complex; and pathways involved in DNA damage/replication checkpoints and cell cycle phase transitions/regulation.

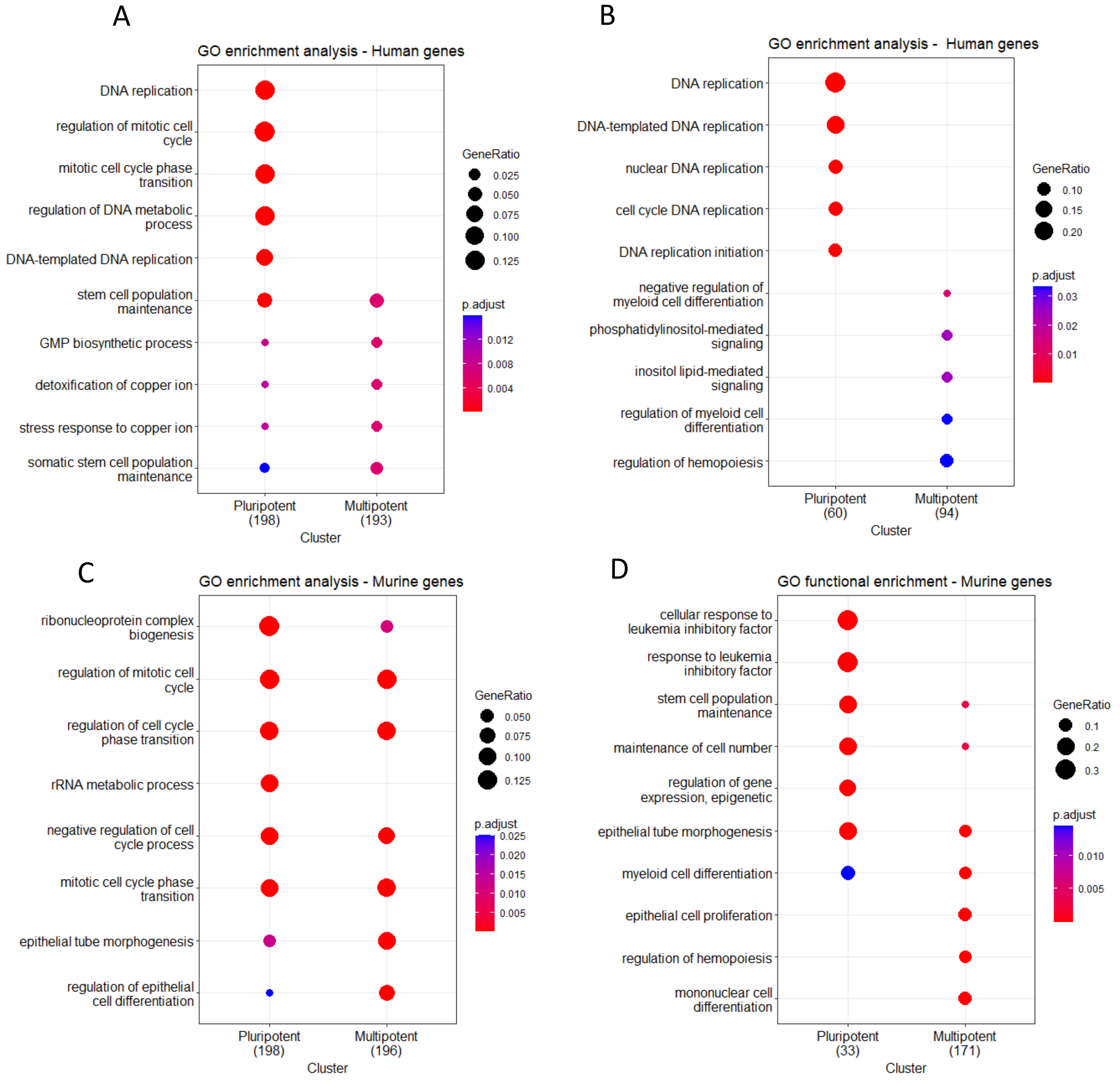

Figure S7 – GO enrichment analysis for genes associated with pluripotency or multipotency stemness signatures.

Dot plots display the results of GO enrichment analysis based on biological processes for (**A**) the 200 genes with the highest score in pluripotency or multipotency gene signatures for human, (**B**) for genes which were specifically associated with either pluripotency or multipotency human stemness signatures i.e. which were included in at least 30% of the pluripotency or multipotency signatures, but not in any of the multipotency or pluripotency signatures for human **(C)** the 200 genes with the highest score in pluripotency or multipotency gene signatures for mouse, and **(D)** for genes which were specifically associated with either pluripotency or multipotency mouse stemness signatures i.e. which were included in at least 30% of the pluripotency or multipotency signatures, but not in any of the multipotency or pluripotency signatures for mouse.

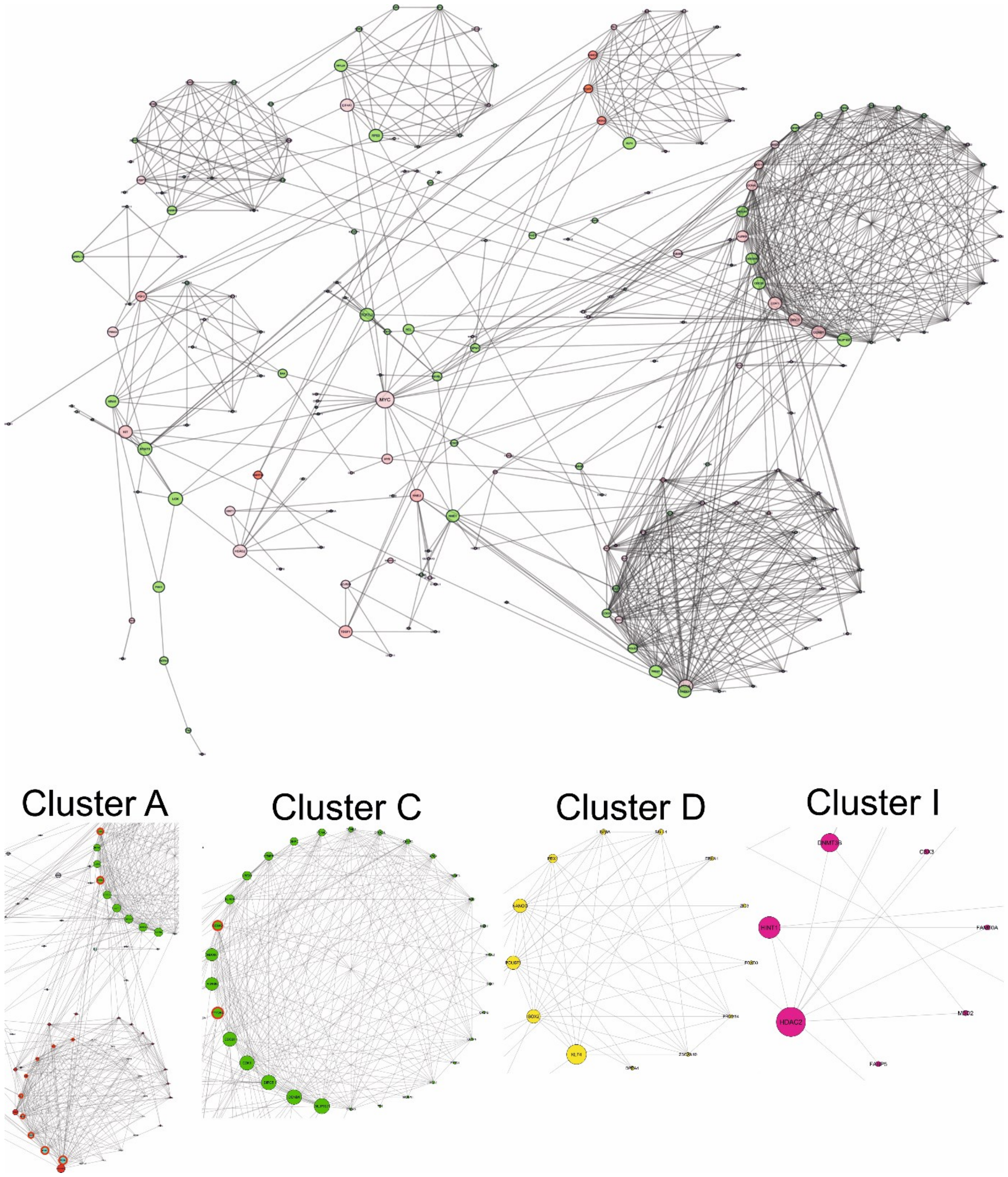

Figure S8 – Complementary figure to Figure 3.

Interactions of proteins corresponding to genes with a minimum score of 3 in the human ranked list are shown. **Top:** Significantly interacting network clusters (p-value < $5 \cdot 10^{-2}$). Nodes of each cluster are disposed in circles according to their betweenness centrality. Red nodes represent genes that belong to the human *ISS* (with score ≥ 4). Edge thickness reflects the interaction confidence score, whereas node size and colour opacity are proportional to node betweenness centrality and the stemness score of the gene, respectively. Nodes without interactions were excluded. Nodes that did not interact with the main network were excluded before the clustering analysis. **Bottom:** Zoomed pictures of clusters in Figure 2B with high clustering significance and high average stemness scores.

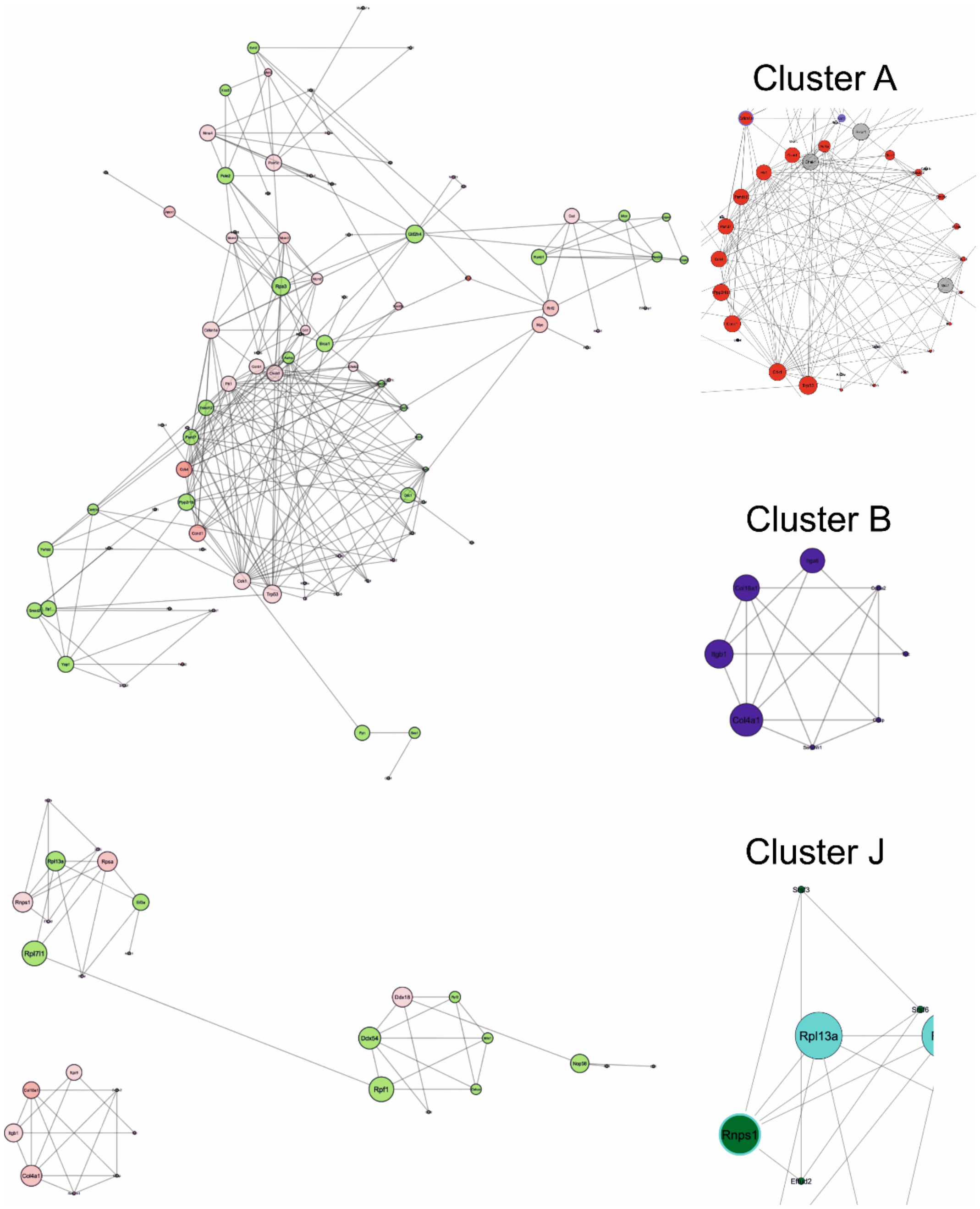

Figure S9 – Complementary figure to Figure 4.

Interactions of proteins corresponding to genes with a minimum score of 6 in the mouse ranked list are shown. **Left:** Significantly interacting network clusters (p-value < $5 \cdot 10^{-2}$). Nodes of each cluster are disposed in circles according to their betweenness centrality. Red nodes represent genes that belong to the mouse *ISS* (with score ≥ 7). Edge thickness reflects the interaction confidence score, whereas node size and colour opacity are proportional to node betweenness centrality and the stemness score of the gene, respectively. Nodes without interactions were excluded. Nodes that did not interact with the main network and were part of a smaller network (with less than four nodes) were excluded before the clustering analysis. **Right:** Zoomed pictures of clusters in Figure 3B with high clustering significance and high average stemness scores.

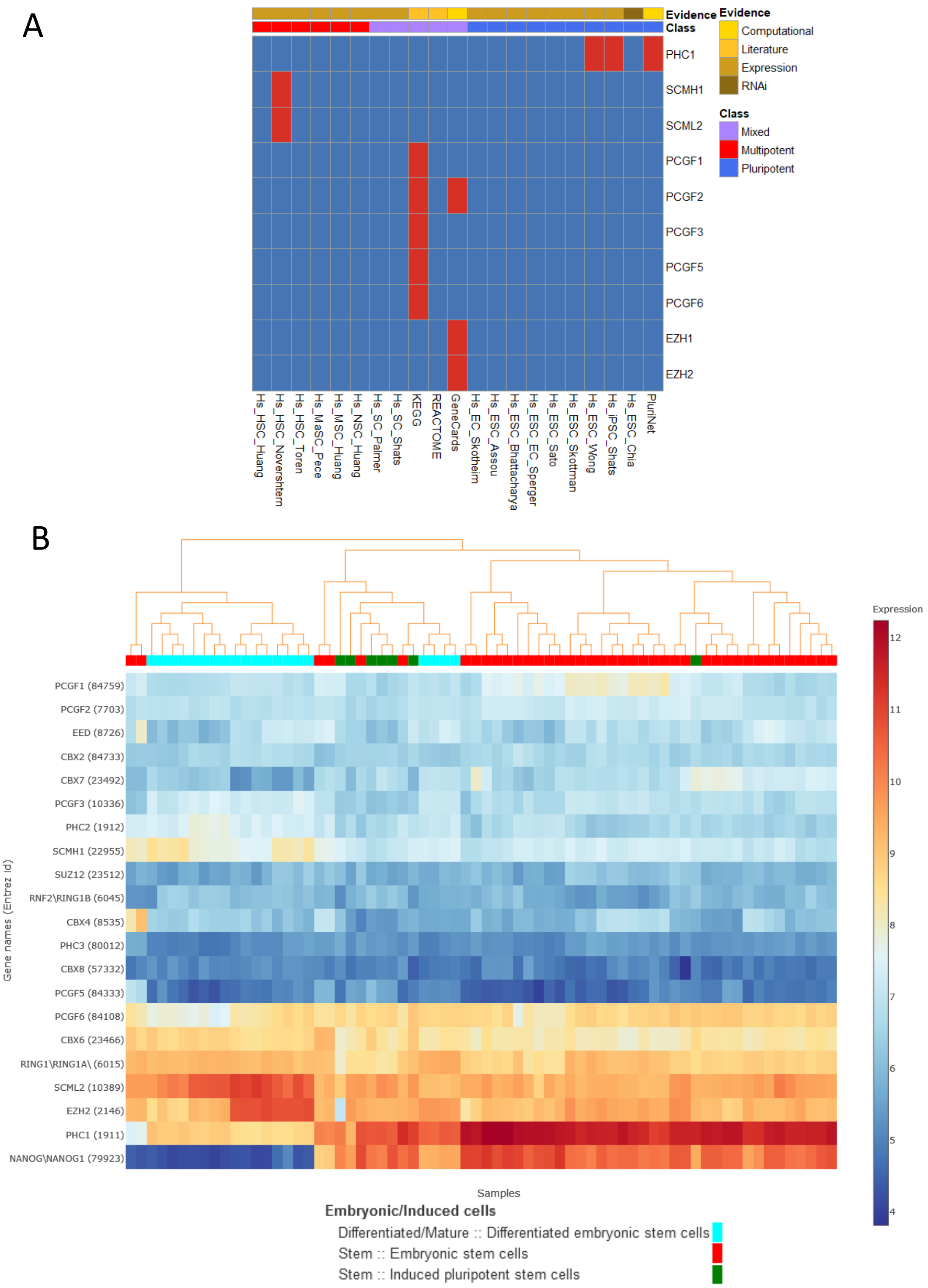

Figure S10 – Stemness association and expression of human Polycomb group (PcG) genes

(A) Checkerboard indicating the inclusion of human PcG genes in stemness signatures. Except *PHC1* and *PCGF2*, PcG genes were included in only one stemness signature (if at all). Most of the present PcG are included because of annotations in KEGG and Genecards. **(B)** Heatmap derived from StemMapper (http://stemmapper.sysbiolab.eu), which displays expression profiles of PcG genes across for ESCs, iPSC and differentiated cells. For comparison, *NANOG* is also displayed in the bottom row. While gene expression changes can be observed for some PcG genes, it is less prominent compared to the sharp drop in expression of *NANOG* in differentiated cells.

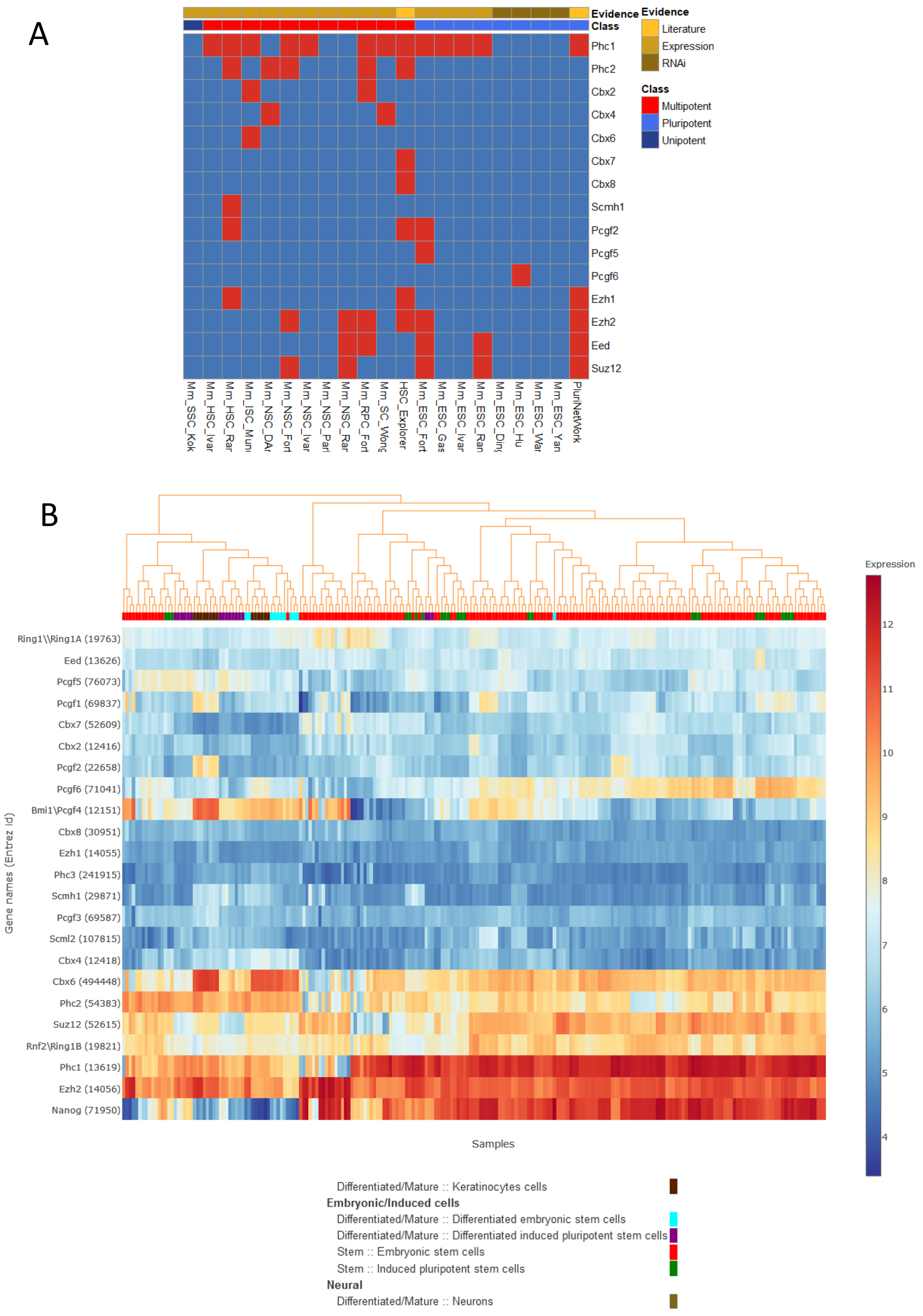

Figure S11 – Stemness association and expression of murine PcG genes

(A) Checkerboard indicating the inclusion of mouse PcG genes in stemness signatures. **(B)** Heatmap derived from StemMapper (http://stemmapper.sysbiolab.eu), which displays expression profiles of PcG genes across for ESCs, iPSC and differentiated cells. For comparison, *Nanog* is also displayed the bottom row.

References

1. Bhattacharya, B.; Miura, T.; Brandenberger, R.; Mejido, J.; Luo, Y.; Yang, A.X.; Joshi, B.H.; Ginis, I.; Thies, R.S.; Amit, M.; et al. Gene Expression in Human Embryonic Stem Cell Lines: Unique Molecular Signature. *Blood* **2004**, *103*, 2956–2964, doi:10.1182/blood-2003-09-3314.

2. Assou, S.; Le Carrour, T.; Tondeur, S.; Ström, S.; Gabelle, A.; Marty, S.; Nadal, L.; Pantesco, V.; Réme, T.; Hugnot, J.-P.; et al. A Meta-Analysis of Human Embryonic Stem Cells Transcriptome Integrated into a Web-Based Expression Atlas. *Stem Cells* **2007**, *25*, 961–973, doi:10.1634/stemcells.2006-0352.

3. Wong, D.J.; Liu, H.; Ridky, T.W.; Cassarino, D.; Segal, E.; Chang, H.Y. Module Map of Stem Cell Genes Guides Creation of Epithelial Cancer Stem Cells. *Cell Stem Cell* **2008**, *2*, 333–344, doi:10.1016/j.stem.2008.02.009.

4. Palmer, N.P.; Schmid, P.R.; Berger, B.; Kohane, I.S. A Gene Expression Profile of Stem Cell Pluripotentiality and Differentiation Is Conserved across Diverse Solid and Hematopoietic Cancers. *Genome Biol* **2012**, *13*, R71, doi:10.1186/gb-2012-13-8-r71.

5. Müller, F.-J.; Laurent, L.C.; Kostka, D.; Ulitsky, I.; Williams, R.; Lu, C.; Park, I.-H.; Rao, M.S.; Shamir, R.; Schwartz, P.H.; et al. Regulatory Networks Define Phenotypic Classes of Human Stem Cell Lines. *Nature* **2008**, *455*, 401–405, doi:10.1038/nature07213.

6. Skotheim, R.I.; Lind, G.E.; Monni, O.; Nesland, J.M.; Abeler, V.M.; Fosså, S.D.; Duale, N.; Brunborg, G.; Kallioniemi, O.; Andrews, P.W.; et al. Differentiation of Human Embryonal Carcinomas *In Vitro* and *In Vivo* Reveals Expression Profiles Relevant to Normal Development. *Cancer Res* **2005**, *65*, 5588–5598, doi:10.1158/0008-5472.CAN-05-0153.

7. Shats, I.; Gatza, M.L.; Chang, J.T.; Mori, S.; Wang, J.; Rich, J.; Nevins, J.R. Using a Stem Cell–Based Signature to Guide Therapeutic Selection in Cancer. *Cancer Research* **2011**, *71*, 1772–1780, doi:10.1158/0008-5472.CAN-10-1735.

8. Sperger, J.M.; Chen, X.; Draper, J.S.; Antosiewicz, J.E.; Chon, C.H.; Jones, S.B.; Brooks, J.D.; Andrews, P.W.; Brown, P.O.; Thomson, J.A. Gene Expression Patterns in Human Embryonic Stem Cells and Human Pluripotent Germ Cell Tumors. *Proceedings of the National Academy of Sciences* **2003**, *100*, 13350–13355, doi:10.1073/pnas.2235735100.

9. Toren, A.; Bielorai, B.; Jacob-Hirsch, J.; Fisher, T.; Kreiser, D.; Moran, O.; Zeligson, S.; Givol, D.; Yitzhaky, A.; Itskovitz-Eldor, J.; et al. CD133-Positive Hematopoietic Stem Cell "Stemness" Genes Contain Many Genes Mutated or Abnormally Expressed in Leukemia. *Stem Cells* **2005**, *23*, 1142–1153, doi:10.1634/stemcells.2004-0317.

10. Huang, T.-S.; Hsieh, J.-Y.; Wu, Y.-H.; Jen, C.-H.; Tsuang, Y.-H.; Chiou, S.-H.; Partanen, J.; Anderson, H.; Jaatinen, T.; Yu, Y.-H.; et al. Functional Network Reconstruction Reveals Somatic Stemness Genetic Maps and Dedifferentiation-Like Transcriptome Reprogramming Induced by GATA2. *Stem Cells* **2008**, *26*, 1186–1201, doi:10.1634/stemcells.2007-0821.

11. Novershtern, N.; Subramanian, A.; Lawton, L.N.; Mak, R.H.; Haining, W.N.; McConkey, M.E.; Habib, N.; Yosef, N.; Chang, C.Y.; Shay, T.; et al. Densely Interconnected Transcriptional Circuits Control Cell States in Human Hematopoiesis. *Cell* **2011**, *144*, 296–309, doi:10.1016/j.cell.2011.01.004.

12. Skottman, H.; Mikkola, M.; Lundin, K.; Olsson, C.; Strömberg, A.; Tuuri, T.; Otonkoski, T.; Hovatta, O.; Lahesmaa, R. Gene Expression Signatures of Seven Individual Human Embryonic Stem Cell Lines. *Stem Cells* **2005**, *23*, 1343–1356, doi:10.1634/stemcells.2004-0341.

13. Sato, N.; Sanjuan, I.M.; Heke, M.; Uchida, M.; Naef, F.; Brivanlou, A.H. Molecular Signature of Human Embryonic Stem Cells and Its Comparison with the Mouse. *Dev Biol* **2003**, *260*, 404–413, doi:10.1016/S0012-1606(03)00256-2.

14. Chia, N.-Y.; Chan, Y.-S.; Feng, B.; Lu, X.; Orlov, Y.L.; Moreau, D.; Kumar, P.; Yang, L.; Jiang, J.; Lau, M.-S.; et al. A Genome-Wide RNAi Screen Reveals Determinants of Human Embryonic Stem Cell Identity. *Nature* **2010**, *468*, 316–320, doi:10.1038/nature09531.

15. Ramalho-Santos, M.; Yoon, S.; Matsuzaki, Y.; Mulligan, R.C.; Melton, D.A. "Stemness": Transcriptional Profiling of Embryonic and Adult Stem Cells. *Science (1979)* **2002**, *298*, 597–600, doi:10.1126/science.1072530.

16. Fortunel, N.O.; Otu, H.H.; Ng, H.-H.; Chen, J.; Mu, X.; Chevassut, T.; Li, X.; Joseph, M.; Bailey, C.; Hatzfeld, J.A.; et al. Comment on " 'Stemness': Transcriptional Profiling of Embryonic and Adult Stem Cells" and "A Stem Cell Molecular Signature" (I). *Science (1979)* **2003**, *302*, 393–393, doi:10.1126/science.1086384.

17. Ivanova, N.B.; Dimos, J.T.; Schaniel, C.; Hackney, J. a; Moore, K. a; Lemischka, I.R. A Stem Cell Molecular Signature. *Science (1979)* **2002**, *298*, 601–604, doi:10.1126/science.1073823.

18. Kokkinaki, M.; Lee, T.-L.; He, Z.; Jiang, J.; Golestaneh, N.; Hofmann, M.-C.; Chan, W.-Y.; Dym, M. The Molecular Signature of Spermatogonial Stem/Progenitor Cells in the 6-Day-Old Mouse Testis1. *Biol Reprod* **2009**, *80*, 707–717, doi:10.1095/biolreprod.108.073809.

19. Guan, K.; Nayernia, K.; Maier, L.S.; Wagner, S.; Dressel, R.; Lee, J.H.; Nolte, J.; Wolf, F.; Li, M.; Engel, W.; et al. Pluripotency of Spermatogonial Stem Cells from Adult Mouse Testis. *Nature* **2006**, *440*, 1199–1203, doi:10.1038/nature04697.
